# How well do modelled routes to school record the environments children are exposed to?: a cross-sectional comparison of GIS-modelled and GPS-measured routes to school

**DOI:** 10.1186/1476-072X-13-5

**Published:** 2014-02-14

**Authors:** Flo Harrison, Thomas Burgoine, Kirsten Corder, Esther MF van Sluijs, Andy Jones

**Affiliations:** 1 UKCRC Centre for Diet and Activity Research (CEDAR), MRC Epidemiology Unit, University of Cambridge School of Clinical Medicine, Box 285 Institute of Metabolic Science, Cambridge Biomedical Campus, Cambridge CB2 0QQ, UK; 2 Norwich Medical School, University of East Anglia, Norwich NR4 7TJ, UK

**Keywords:** Route to school, Food environment, Physical activity environment, Geographic information systems, Global positioning systems

## Abstract

**Background:**

The school journey may make an important contribution to children’s physical activity and provide exposure to food and physical activity environments. Typically, Geographic Information Systems (GIS) have been used to model assumed routes to school in studies, but these may differ from those actually chosen. We aimed to identify the characteristics of children and their environments that make the modelled route more or less representative of that actually taken. We compared modelled GIS routes and actual Global Positioning Systems (GPS) measured routes in a free-living sample of children using varying travel modes.

**Methods:**

Participants were 175 13-14 yr old children taking part in the Sport, Physical activity and Eating behaviour: Environmental Determinants in Young people (SPEEDY) study who wore GPS units for up to 7 days. Actual routes to/from school were extracted from GPS data, and shortest routes between home and school along a road network were modelled in a GIS. Differences between them were assessed according to length, percentage overlap, and food outlet exposure using multilevel regression models.

**Results:**

GIS routes underestimated route length by 21.0% overall, ranging from 6.1% among walkers to 23.2% for bus users. Among pedestrians food outlet exposure was overestimated by GIS routes by 25.4%. Certain characteristics of children and their neighbourhoods that improved the concordance between GIS and GPS route length and overlap were identified. Living in a village raised the odds of increased differences in length (odds ratio (OR) 3.36 (1.32-8.58)), while attending a more urban school raised the odds of increased percentage overlap (OR 3.98 (1.49-10.63)). However none were found for food outlet exposure. Journeys home from school increased the difference between GIS and GPS routes in terms of food outlet exposure, and this measure showed considerable within-person variation.

**Conclusions:**

GIS modelled routes between home and school were not truly representative of accurate GPS measured exposure to obesogenic environments, particularly for pedestrians. While route length may be fairly well described, especially for urban populations, those living close to school, and those travelling by foot, the additional expense of acquiring GPS data seems important when assessing exposure to route environments.

## Background

The environments within which children live and play are potentially important drivers of their health related behaviours [1]. There has been much recent interest in how the characteristics of home neighbourhoods influence diet [2] and physical activity [3] through, for example, the availability of play spaces, access to both healthy and unhealthy foods, and the provision of roads and footpaths for active travel. However, researchers are increasingly recognising how environments outside those immediately proximal to the home may also be important determinants of these health behaviours. For instance, children spend a large amount of time at school, and so the characteristics of school neighbourhoods are also now seen as important locations [4], as are routes between home and school. Interest in routes is often associated with work on the determinants of active travel [5-7], but some research is beginning to look at the opportunity travel to school presents for access to food environments [8,9] and physical activity facilities [4].

Past work has typically relied on assessments of route characteristics based on parents’ and children’s perceptions [10], or using a geographic information system (GIS) to objectively characterise a modelled route to school based on the assumption that children will take the shortest route [4-9]. Indeed, until recently these methods have been the only options available to researchers wishing to investigate the location and characteristics of children’s routes to and from school. However, with the current availability of small, low-cost global positioning system (GPS) devices, it is now possible to record and characterise the actual routes children take.

The GIS approach to modelling routes has some advantages. Assuming home and school locations are known, routes can be calculated quickly for large numbers of children. Routes may also be modelled at any point during the research process; for post hoc analyses, or to predict possible changes in routes to school that may be brought about by environmental changes, such as the building of a new school. However, it is not clear how well these modelled routes reflect those actually taken by children, nor how well they describe the environments children pass through on their way to and from school.

There are a number of reasons why shortest routes may not accurately reflect those actually taken. For instance, children may prefer alternatives that offer safer or more attractive paths, or opportunities to visit other destinations on the way. Additionally the digital road networks used in the GIS to predict shortest routes may not include all paths available, especially informal pedestrian short-cuts. Although some work has compared GIS modelled routes with those measured by GPS devices [11,12], the samples assessed have tended to be limited by small numbers, and the inclusion of people travelling by just a single mode, or set in just one urban location. Differences in routes have been assessed by a variety of metrics relating to urban design only such as land use mix, presence of busy streets, and street connectivity. Duncan & Mummery [12] found consistency in route length between GIS and GPS measures among children walking to school, but some differences in their exposure to busy streets, with GPS routes typically following a greater proportion of quieter roads. Among adults, differences in the assessment of built environment characteristics of GIS and GPS routes were found to be dependent on the specific measure and route buffer size used [11]. However, that study included only 29 commuting routes to work, of which 20 were made by car.

It remains unclear how well GIS routes to school match those measured by GPS for children travelling by modes other than walking, and for children living in non-urban locations. Furthermore, past work has tended to focus on environmental measures relating to walkability and the built environment, but the impact of route modelling on the assessment of exposure to the food environment is unclear. Childhood obesity and dietary intake have been associated with the availability of foods through the presence of food outlets within home and school neighbourhoods [13,14] and the journey to and from school may particularly represent an opportunity for children to interact with such environments [15]. Several studies have sought to explore exposure to the food environment during school travel times by examining associations between the number of food outlets passed on a modelled route between home and school and dietary intake [9] and weight status [4,8], so testing the accuracy of this modelled exposure is timely.

The aim of this paper is therefore to assess how the use of GIS and GPS routes affect the assessment of environmental exposure measures, and to identify which characteristics of children and their environments make the modelled route more or less representative of that actually taken. The work will assess the circumstances under which a GIS modelled route may provide an adequate definition, and when it is likely to be most important to obtain GPS data. We expand on past work by using a broad sample of children using varying means of transportation and living in diverse urban–rural settings in the county of Norfolk, UK. Additionally we compare GIS and GPS routes not only in terms of their length and shape, but also in how they characterise children’s food environment exposures, an as yet under-investigated measure.

## Methods

### Study design and recruitment

Data for these analyses came from the third phase of the SPEEDY study (Sport, Physical activity and Eating behaviour: Environmental Determinants in Young people). SPEEDY is a population-based longitudinal cohort study, investigating factors associated with physical activity and dietary behaviour among children attending schools in the county of Norfolk, UK. Details of participant recruitment and study procedures at baseline data collection [16] and at four-year (third phase) follow up [17] are described elsewhere.

In 2007, 2064 Year 5 (aged 9–10 years) pupils were recruited from 92 Norfolk primary schools, selected to maintain urban/rural heterogeneity. The third phase of SPEEDY data collection was a four-year follow-up in the summer term of 2011. Of the 56 schools attended by SPEEDY participants, 19 were selected for GPS measurements. The selection of schools was made to maximise heterogeneity in terms of both urban/rural status and area socio-economic status, and to include schools with high participant numbers. The analyses presented here utilise data from 175 Year 9 children (aged 13–14 years) who returned GPS devices and questionnaires (36% of all SPEEDY participants, and 77% of all SPEEDY participants at schools selected for GPS measurements). Prior to participation pupils returned consent forms signed by themselves and a parent, and ethical approval for the study was obtained from the University of East Anglia research ethics committee (approval number 2010/2011 – 26).

### Study data collection

Participating children and their parents were asked to complete questionnaires about themselves, and their beliefs and practices around diet and physical activity. From their responses, basic demographic information, including sex, and household income, were obtained along with their usual mode of travel to school, for which response options were: car, bus, bicycle or on-foot. Participants’ home addresses were provided on consent forms and home urban/rural status (being classed as either Urban, Town and fringe (semi-urban) or Village, hamlet and isolated dwelling (rural)) [18], was defined by the census area (lower super output area) the address fell within. These are geographic areas used for the collection and publication of small area statistics from the UK census, each containing approximately 1500 residents. Schools were also assigned an urban/rural status based on their address, so that the urbanicity of a participants school relative to their home could be assessed (e.g. a child living in a village location, but attending a ‘Town & Fringe’ school goes to a ‘more urban’ school).

Home addresses were geocoded using Ordnance Survey’s (OS) Address Layer 2 [19], a database of all UK addresses and their geographic location at the building level. A school grounds audit, adapted from that used in the primary schools participating at baseline [20] was undertaken at all secondary schools participating in the third phase of the study. The audit included the identification of all school entrances, which were recorded on a paper map, and later digitised in a GIS (ArcGIS v10.1 [21]). Secondary schools may have large grounds with multiple access points. Identification of all entrances therefore enables the modelling of routes to school more accurately than if a single point were used to represent the school.

All consenting participants at the schools selected for GPS measurements were visited at school by researchers to fit GPS devices, which were returned to school one week later. Participating children were asked to wear a Qstarz BT-Q1000XT waist-mounted GPS device for seven consecutive days. These devices are accurate to 3 m [22] and were set to record location at 10-second intervals. Participants were instructed to charge the device’s battery every night, and to put it on first thing in the morning and wear it all day until they went to bed. They were also asked to remove the device while participating in any aquatic activities.

### Route definitions

GIS routes were modelled assuming the shortest distance route along the road network between participant home and their nearest school entrance as identified in the school grounds audit. The OS Integrated Transport Network (ITN) [23], was employed as the road network. ITN includes all motorways, A roads, B roads, minor roads, local streets and private roads, but not footpaths. Routes were modelled using the Network Analyst extension for ArcGIS 10.1 [21].

For GPS routes, all GPS data recorded for the periods 07:30–09:00 and 15:00–16:30 on school days were initially extracted and manually examined to determine the points making up participants’ travel between home and school. Where routes to or from school were not completed within these times, additional points covering the periods 06:00–10:30 and 13:00–18:00 were extracted. If routes between home and school could still not be determined within these times, the participant was deemed not to have travelled to or from school during that period. All children with at least one route to or from school were included in these analyses.

All routes were manually cleaned, in that the lead author loaded each individual set of GPS points (a separate file for each participant/day/session) into a GIS and visually inspected them. This enabled firstly the extraction of just those points that constituted the route to/from school, which started/finished with the first/last point within 20 m of the school/home grounds, and secondly the identification of points affected by GPS drift. This process was necessary as the positional accuracy of data recorded by a GPS device is dependent on the number of satellites it can connect to. When the device is first turned on, it can take some time to acquire a good signal, and during this time the points recorded may be somewhat dispersed. The same effect can also arise in urban areas when satellite signals are blocked by tall buildings. Possibly due to the largely rural, low-lying nature of the study setting, and improvement in GPS technologies, signal drop-out was not observed to be a problem, and signal drift resulted in the loss of less than 1% of recorded points.

Many routes appeared to include stops at various locations between home and school (e.g. shops, other houses, parks), and all points recorded during these stops were removed from the route for the calculation of route length. All points forming each individual route were joined to create a line feature using Geospatial Modelling Environment [24].

### Route characterisation

For each GIS and GPS route we determined a number of characteristics, which have previously been shown to differ between GIS and GPS routes (e.g. busy roads [12]), or which have been used to assess exposure to food and physical activity environments in studies using GIS-modelled routes [4,8,9]. We calculated the length of each route, the percentage falling on ‘busy’ (A and B) roads, and, for GPS routes, percentage not on part of our road network. GPS points were joined to the road network, and assigned the characteristics of the nearest road segment. Any point not falling within 20 m of the network was classified as ‘not on road’. This distance was felt sufficient to allow for average road widths and mislocation due to poor satellite signal, while minimising misclassification, and the erroneous linking of points recorded on paths not included in our digital network to roads.

The location of food outlets in our study area were obtained from 12 district and city councils (local administrative authorities) in Norfolk, Suffolk and Lincolnshire in January 2012. Outlets were classified based on a six point scheme (takeaways, restaurants, convenience stores, supermarkets, specialist stores, and cafes) [25] derived from the 21-point scheme developed by Lake et al. [26]. Takeaways and convenience stores were grouped as ‘unhealthy’ food outlets. The locations of physical activity facilities were derived from OS Points of Interest (POI) [27], and included all locations classed as sports centres or community centres, a definition we have used previously [4]. In order to assess the availability of food outlets and physical activity facilities on both GIS and GPS routes, we generated 100 m buffers around each route, and counted the number of food outlets and physical activity facilities within them. One-hundred metre buffers are intended to measure the area accessible that surrounds the route, and is a measure that has been used previously [4,5,8].

### Analysis

Simple comparisons were made between GIS and GPS routes in terms of length, percentage of route on a busy road, total number of food outlets passed, number of unhealthy food outlets passed and number of physical activity facilities passed. Comparisons were made for all routes, and for those made by children usually using different modes of transport to school (car, bus, bicycle, and on foot). As a result of the skewed distributions, comparisons of GIS and GPS values were made using Wilcoxon paired rank tests.

A modelling approach was then taken to determine the characteristics of participants and their environments that were associated with modelled and actual route differences. We selected three outcome variables to model the correlates of differences between GIS and GPS routes in more detail. First, differences in route length were calculated as length of GPS route minus length of GIS route. Second, route shape differences were assessed as the percentage of the GPS route falling within 50 m of the GIS route. The 50 m buffer was used to allow paths parallel to roads to be treated as being the same. Third, we calculated the difference in the number of food outlets passed on each route (GPS food outlets minus GIS food outlets). These three measures were chosen as they represent three different aspects of route characteristics potentially useful in future research. Assessment of route length may be important in the assessment of physical activity or travel mode choice, while determining exactly which way a person has gone (percentage of GPS route falling within GIS route buffer) may be important for assessing what environments they are exposed to. The third measure (difference in exposure to food outlets) tests whether taking a different route actually impacts a given environmental exposure, specifically one modelled in several recent studies [4,8,9].

**Figure 1:**
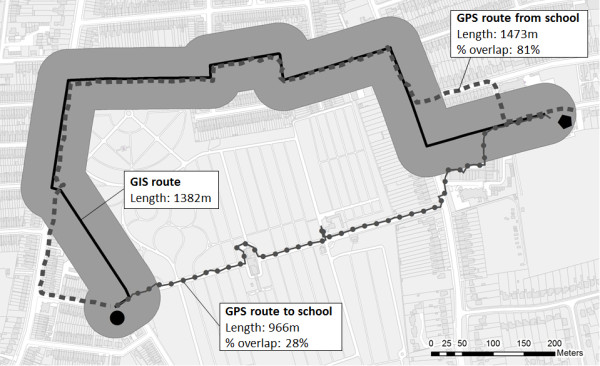
**Example of GIS and GPS routes between home (black circle) and school (black pentagon).** The 50 m buffer around the GIS route is used to assess overlap with GPS routes. NB to protect participant anonymity these are simulated data. © Crown Copyright/database right 2013. An Ordnance Survey/EDINA supplied service.

Examples of the first two variables are shown in Figure 1. Here, for a fictional participant, GPS routes to and from school are shown along with the modelled GIS route. In this example the GPS route home from school is longer than the GIS route, but follows a largely similar path, as reflected by the high percentage overlap value. The GPS route to school is shorter and takes a completely different path to that modelled in the GIS. Although the GIS models the shortest route along the road network, the GPS measured route may be shorter if the participant has used pedestrian paths or short cuts not present in the digital network.

Multilevel models allowing for clustering of routes within individuals within schools (3-level models) were used to quantify the correlates of each of the three outcome metrics studied. Explanatory variables that were statistically significant (p < 0.05) in univariate models were included in a multivariable model. A backwards step-wise modelling approach was employed, removing non-significant variables (p ≥ 0.05) step-wise to produce a best fit model. As, all else remaining equal, we might expect difference in length to increase the further children live from school, straight-line distance between home and school was included as a co-variate in all length difference models. The distributions of both the differences in length and percentage overlap variables were not normal, so both were categorised into tertiles and analysed using ordinal logistic regression models, after testing the proportional odds assumption using the Brant test on single level versions of the models. Outcomes are presented as odds ratios for moving up a tertile category. Differences in food outlet exposure were modelled using multilevel linear regression. Given that negative values are plausible in this variable (i.e. there are more food outlets on the GIS route), coefficients represent an increasing tendency towards more food outlets on the GPS route relative to the GIS route, rather than higher absolute numbers on the GPS route.

Variance partition coefficients (VPC) were calculated for the best fit models to determine the proportion of unexplained variance in the outcomes lying at each level (trip, individual, school attended) in the model hierarchy. VPCs were calculated by dividing the residual variance at each level, by the total residual variance for all three levels.

All analyses were undertaken in Stata version 11 [28].

## Results

The sample of 175 participants provided GPS data on 1191 routes; 528 to school, and 663 from school. There were a median of 7 routes per child (Inter Quartile Range (IQR) 5–9) and a median of 5 children per school (IQR 4–13). Table 1 shows the characteristics of the participants and their routes. There were no statistically significant differences between those included in these analyses and all SPEEDY 3 participants in terms of these demographic characteristics. Roughly equal numbers of boys and girls were included in this sample. Almost 40% of participants usually travelled to school by bus, and only 8 participants (4.6%) reported usually travelling by bicycle.

**Table 1:** Participant characteristics

	**Participants**		**GPS routes**	
	**N**	**%**	**N**	**%**
Total/All	175		1191	
Sex				
Girl	89	50.86%	614	51.55%
Boy	86	49.14%	577	48.45%
How do you usually travel to school?				
By car	41	23.43%	262	22.00%
By bus or train	68	38.86%	475	39.88%
By bicycle	8	4.57%	62	5.21%
On foot	58	33.14%	392	32.91%
Home location				
Urban	57	32.57%	367	30.81%
Town & Fringe	44	25.14%	300	25.19%
Village, Hamlet & Isolated Dwelling	74	42.29%	524	44.00%
What is your annual household pre-tax income?				
Up to £10,000	17	9.71%	96	8.06%
Over £10,000 to £30,000	40	22.86%	284	23.85%
Over £30,000 to £50,000	49	28.00%	341	28.63%
Over £50,000 to £70,000	30	17.14%	184	15.45%
Over £70,000	10	5.71%	78	6.55%
I do not wish to share this information	28	16.00%	203	17.04%

NB No significant differences between SPEEDY 3 participants with GPS and all SPEEDY 3 participants in terms of these demographic characteristics.

Table 2 provides information on the routes measured by GPS, overall, and by usual travel mode. On average, GPS route lengths were longest for bus travellers followed by those travelling by car, bike and on foot, respectively. A small percentage of the GPS routes did not appear to fall on the road network used to model GIS routes. The overall median percentage not on the road network was 0.3%, but this was considerably higher for those travelling by foot (median 4.8%, IQR 0% -10.9%). In terms of overlap with GIS routes, the distribution was flat, with similar numbers of GPS routes across values 0% to 100%. There were slight differences in the distribution of percentage overlap between mode groups. Among cyclists the median was lower (41.4%), but this category had the highest 25th and 75th centile values.

**Table 2:** GPS route characteristics by usual mode of travel to school

**Median (IQR; 25th centile-75th centile)**	**Total**	**By car**	**By bus**	**By bicycle**	**On foot**
N	1191	262	475	62	392
GPS route length (km)	4.56 (6.78; 1.57 - 8.35)	4.14 (5.98; 2.44 - 8.42)	7.99 (5.27; 6.21 - 11.48)	2.02 (2.90; 1.14 - 4.04)	1.39 (0.93; 0.92 - 1.82)
Percentage of GPS route not on a road	0.31 (4.71; 0–4.71)	0.34 (3.09; 0–3.09)	0.04 (0.65; 0–0.65)	0 (6.25; 0–6.25)	4.82 (10.88; 0–10.88)
Percentage of the GPS route that falls within the 50 m GIS route buffer	54.43 (57.37; 26.47 - 83.84)	57.9 (61.48; 23.28 - 84.76)	52.71 (52.91; 25.09 - 78)	41.42 (57.77; 32.55 - 90.32)	56.33 (61.75; 27.8 - 89.55)

Differences in environmental measures between GIS and GPS routes are shown in Table 3. On average, considering all routes, GPS routes were 21.0% longer than those modelled in the GIS. Differences in length varied considerably by mode, but even the mode with the smallest difference (walking) showed a statistically significant underestimation of route length when using GIS (difference 6.1%, p < 0.01). Patterns for the other environmental variables were less consistent. Overall there was no statistically significant difference in exposure to busy roads nor to total food outlets between GIS and GPS routes, although significantly more unhealthy food outlets and physical activity facilities were passed on GPS routes compared to their GIS counterparts. However, exposure differences varied across travel modes. Typically, differences were less, or even negative (i.e. exposure greater on GIS routes than GPS) for pedestrians and cyclists, and higher for bus and car users. In this sample GIS routes appeared to significantly overestimate exposure to food outlets for pedestrians in particular.

**Table 3:** Differences in route characteristics between GIS and GPS routes for all routes, and by usual mode of travel to school

		**GIS mean**	**GPS mean**	**Difference (GPS-GIS)**	**95% CI for diff**		**Difference as% of GPS value**	**p**^**1**^	**p**^**2**^
					**Lower**	**Upper**			
Length (m)	Total N = 1191	4532.4	5739.5	1207.1	1031.3	1383.1	21.03%	**<0.01**	
	By car N = 262	5219.2	6672.0	1452.8	922.7	1982.9	21.77%	**<0.01**	**<0.01**
	By bus N = 475	6936.7	9032.3	2095.6	1818.1	2373.0	23.20%	**<0.01**	
	By bicycle N = 62	2486.7	2868.4	381.7	173.5	589.8	13.31%	**<0.01**	
	On foot N = 392	1483.4	1580.4	97.0	−61.1	255.3	6.14%	**<0.01**	
% of route on busy (A&B) roads	Total N = 1191	25.816	26.170	0.354	−1.042	1.750	1.35%	*0.925*	
	By car N = 262	34.178	30.477	−3.701	−6.998	−0.404	−12.14%	*0.073*	**<0.01**
	By bus N = 475	30.240	35.588	5.348	3.003	7.692	15.03%	**<0.01**	
	By bicycle N = 62	23.253	18.829	−4.423	−12.314	3.467	−23.49%	*0.915*	
	On foot N = 392	15.270	13.039	−2.230	−4.015	−0.446	−17.10%	**0.010**	
Number of food outlets on route	Total N = 1191	6.205	6.530	0.325	−0.134	0.784	4.98%	*0.938*	
	By car N = 262	7.767	9.099	1.332	0.040	2.624	14.64%	*0.359*	**<0.01**
	By bus N = 475	6.309	7.368	1.059	0.283	1.835	14.37%	**<0.01**	
	By bicycle N = 62	4.323	3.726	−0.597	−2.069	0.876	−16.02%	*0.448*	
	On foot N = 392	5.332	4.240	−1.092	−1.587	−0.597	−25.75%	**<0.01**	
Number of unhealthy food outlets on route	Total N = 1191	2.783	2.934	0.150	−0.071	0.371	5.12%	**0.016**	
	By car N = 262	3.408	3.901	0.492	−0.107	1.091	12.62%	*0.546*	**<0.01**
	By bus N = 475	2.720	3.139	0.419	0.027	0.810	13.35%	*0.178*	
	By bicycle N = 62	2.323	2.306	−0.016	−1.002	0.970	−0.70%	*0.526*	
	On foot N = 392	2.515	2.138	−0.378	−0.579	−0.176	−17.66%	**<0.01**	
Number of physical activity facilities on route	Total N = 1191	1.823	2.251	0.428	0.322	0.535	19.02%	**<0.01**	
	By car N = 262	1.656	2.653	0.996	0.707	1.285	37.55%	**<0.01**	**<0.01**
	By bus N = 475	2.423	2.844	0.421	0.267	0.575	14.80%	**<0.01**	
	By bicycle N = 62	0.661	1.371	0.710	0.469	0.950	51.76%	**<0.01**	
	On foot N = 392	1.390	1.403	0.013	−0.154	0.179	0.91%	*0.668*	

All difference are GPS - GIS therefore positive values = more on GPS route, and negative values = more on GIS route. p^1^ p for differences between GIS and GPS values within group (Wilcoxon paired rank test). p^2^ p for difference in differences between groups (Kruskal-Wallis equality-of-populations rank test). for all p values, **bold** font indicates p < 0.05, *italic* font indicates p > 0.05.

Table 4 shows the regression models obtained for differences in route length and percentage overlap. Travel mode and home location were significant predictors of differences in route length after adjustment for distance between home and school. Longer GPS routes relative to GIS routes were seen for bus travellers (OR 11.32, 95%CI 4.96-25.86) and those living in villages (OR 3.49, 95%CI 1.89-6.49), while the opposite was seen for walkers (OR 0.06, 95%CI 0.03-0.13). These associations, although slightly attenuated, remained in the best fit model, along with straight-line distance; every additional kilometre between home and school increased the odds of moving up a tertile of length difference by 1.37.

**Table 4:** Results from multilevel ordinal logistic regression models of differences in route lengths, and percentage overlap between GIS and GPS routes

		**Differences in Length**								**% of GPS route within 50 m GIS route buffer**							
		**Adjustment for straight-line distance only**				**Best fit**				**Univariate associations**				**Best fit**			
			**95% CI**				**95% CI**				**95% CI**				**95% CI**		
		**OR**	**Lower**	**Upper**		**OR**	**Lower**	**Upper**		**OR**	**Lower**	**Upper**		**OR**	**Lower**	**Upper**	
Straight-line distance		2.25	2.02	2.52	**	1.37	1.16	1.62	**	1.11	1.01	1.20	*	0.82	0.72	0.94	**
Journey type (ref to sch)	From school	1.14	0.85	1.54						0.76	0.56	1.02					
Sex (ref male)	Female	0.41	0.23	0.74	*					3.44	2.11	5.62	**				
Travel mode (ref car)	Bus	11.32	4.96	25.86	**	8.32	3.08	22.50	**	1.02	0.54	1.92		1.93	1.00	3.72	
	Bike	2.40	0.88	6.54		0.55	0.17	1.80		1.33	0.45	3.90		2.79	1.07	7.31	*
	Foot	0.06	0.03	0.13	**	0.15	0.06	0.36	**	0.54	0.29	1.01		1.42	0.63	3.19	
Home location (ref urban)	Town & Fringe	1.54	0.80	2.96		0.75	0.34	1.64		0.42	0.25	0.72	**	0.19	0.11	0.35	**
	Village etc.	3.49	1.87	6.49	**	4.62	1.74	12.22	**	0.77	0.47	1.29		0.26	0.10	0.74	*
School location relative to school (ref no diff)	School less urban	0.49	0.15	1.63						0.08	0.04	0.17	**	0.98	0.35	2.79	
	School more urban	2.27	1.30	3.96	**					3.26	2.03	5.26	**	3.98	1.49	10.63	**
Variance partition coefficients						Variance		VPC^a^						Variance		VPC^a^	
Level 1 (Route)						3.29		32.10%						3.29		16.72%	
Level 2 (Participant)						6.24		60.91%						12.41		63.09%	
Level 3 (School)						0.72		7.00%						3.97		20.19%	

The distributions of both outcome variables did not allow for linear modelling, so both were banded into tertiles. Outcomes are presented as odds ratios for moving up a tertile category. Difference in Length Tertiles; GIS longer, GPS up to 600 m longer, GPS >600 m longer.% overlap tertiles: 0.1%-33%, >33%- < 73%, ≥73%-100%. *p < 0.05, **p < 0.01, ^a^Variance Partition Coefficient.

The models for percentage overlap between GPS and GIS routes show some similarities to the difference in length models. Living further from school decreased percentage overlap (per km OR 0.82 95%CI 0.72-0.94), as did living in a village location (and also a town/fringe location; ORs and 95% CI 0.26, 0.10-0.74 and 0.1, 0.11-0.35 respectively). Relative to travel by car, all other modes of travel increased percentage overlap (OR 2.79 95%CI 1.07-7.31), although this was only statistically significant among cyclists. Additionally, attending a school in a location more urban than the home location also increased percentage overlap (OR 4.00, 95%CI 1.50-10.63).

Results for the model predicting difference in food outlet exposure (Table 5) were less revealing. The only variable to be significantly associated with difference in food outlet exposure was whether the GPS route was to or from school. Routes home from school had an average of 1.5 more food outlets on the GPS route compared to the GIS route.

**Table 5:** Results from multilevel linear regression models of differences in food outlet exposure between GIS and GPS routes

		**Univariate**			
			**95% CIs**		
		**B**	**Lower**	**Upper**	
Straight-line distance (km)		0.04	−0.21	0.30	
Journey type (ref to school)	From school	1.53	0.80	2.27	**
Sex (ref male)	Female	0.35	−1.37	2.08	
Travel mode (ref car)	Bus	0.59	−1.65	2.84	
	Bike	−1.66	−5.92	2.60	
	Foot	−1.74	−4.07	0.59	
Home location (ref urban)	Town & Fringe	−1.78	−4.13	0.58	
	Village etc.	0.55	−1.54	2.64	
School location relative to school (ref no diff)	School less urban	1.21	−2.89	5.31	
	School more urban	1.58	−0.21	3.37	
Variance partition coefficients		Variance		VPC^a^	
Level 1 (Route)		42.24		60.44%	
Level 2 (Participant)		27.29		39.06%	
Level 3 (School)		0.35		0.50%	

**p < 0.01, ^a^Variance Partition Coefficient.

Variance partition coefficients (VPCs) for the best fit models show some differences. For percentage overlap (Table 4), 63% of the variance occurred at the participant level and 17% at the route level, indicating a tendency for similar values for the different routes made by the same individual. In contrast, VPC values for the food outlet differences model (Table 5) were 60% at the route level and 39% at the individual, indicating greater within-person variance in food outlet exposure.

## Discussion

In our sample, statistically significant differences in environmental exposures were found between GIS and GPS routes. This was particularly evident among pedestrians for whom GIS routes underestimated true route length, and overestimated exposure to busy roads, total food outlets and unhealthy food outlets. Our results suggest that while a GIS route may provide a reasonable proxy measure of route length, caution should be exercised in the assessment of environmental exposure.

GIS routes underestimated route length by an average of 21%. Underestimation was less severe for active travellers, but was still statistically significant. Living further from school, travelling by bus and living in rural locations were all associated with greater differences in length between GIS and GPS routes. GIS estimates of route length for children with these characteristics are therefore likely to be least reliable. These finding may have some impact on studies attempting to estimate physical activity accrued during travel to school. Although the mean difference of 97 m for those travelling on foot may represent only a small difference in potential physical activity, such differences may also be important for work attempting to identify distance thresholds for different modes, or for work such as that of Singleton (2014) attempting to estimate CO_2_ emissions from school commutes, where a 1 km difference in route length for car drivers may make a significant difference [29].

In terms of the specific environmental exposures we investigated, the general trend seemed to be that GIS routes overestimated exposure for active travellers, and underestimated for bus and car users. The impact of underestimation on environmental exposures in bus and car users is not necessarily clear, as their actual exposure will be dependent on their exiting their vehicles, and further research on this behaviour is required. In a finding similar to that of Duncan & Mummery [12], the study of GPS routes revealed a preference for quieter roads among walkers; the length of the route along a busy road was 17% lower on GPS routes compared to GIS routes. This trend was also apparent, although not statistically significant, among cyclists.

Given that walkers and cyclists potentially have greater opportunity to access the facilities they pass en route, accurate assessment of their exposure is important. Although the best fit model of percentage overlap indicated that certain characteristics of children and their environments (living closer to school, travelling by bike, living in an urban location, or attending a school in a more urban location) increased the likelihood that the GIS route more accurately represented that taken, the same factors were not associated with differences in the environmental exposure variable, food outlet exposure, as examined in regression models. Mean food outlet exposure ranged from 4–9 outlets on a route, according to travel mode, so it is possible that a relatively small deviation from the modelled route could result in a proportionally large difference in food outlet exposure, especially if outlets are clustered and a relatively large number may be passed in a short distance.

The only variable we found to be significantly associated with differences in food outlet exposure was whether the route was to or from school. Disparities in estimated exposure were greater by an average of 1.5 outlets for journeys home compared with those to school. It may be important to consider differing environmental exposures on routes to and from school in future work. Certainly, if GPS are being used to record routes, efforts should be made to include travel in both directions. It may be that during the period after school children have more time to deviate from a direct route, and therefore greater exposure to the school and route foodscapes can occur. Indeed, in this sample, mean food outlet exposure was 5.6 outlets across GPS routes to school, and 7.2 outlets across GPS routes from school.

While our results indicate that GIS modelled routes do not capture actual environmental exposures particularly well, the use of GPS data is also not without issue. Chaix et al. [30] argue that as GPS devices measure only where individuals have been, and not the environment they have the potential to use, the causality between environmental exposure and health behaviour is obscured. However, we believe that further use of GPS route measurement, coupled with GIS derived ‘potential environments’ and behavioural surveys and interviews may allow this issue to be unpicked, for example potentially examining how and why a child may deviate from the shortest route home to access food outlets, and thereby improving our understanding of how environments and behaviours interact.

In addition to this conceptual issue, the use of GPS data also raises questions about data representativeness. We modelled routes separately for each day and session (to or from school), giving up to 10 routes for each participant. Further research is needed to better understand how many routes may need to be recorded to assess habitual exposure. However, given the differences we found in variance partition when modelling percentage overlap (a general measure of path concordance) and food outlet differences (a specific environmental exposure measure), the number of routes required may vary according to the exposures being investigated.

This study has several strengths and weaknesses. In terms of strengths we included a large number of objectively measured GPS routes from participants living in a range of urban and rural settings. Participants travelled by different modes, and were recorded over multiple days. Secondary school-aged children such as those studied here are likely to travel independently to and from school [31], and therefore take routes of their own choosing.

While processing tools exist for the identification of trips within GPS data [32], it is not clear how successful the automated identification of routes to school may be, especially as they may be composed of multiple ‘trips’ if the individual has stopped along the way. To prevent potential errors as a result of trip identification automation, we manually identified routes between home and school from the GPS data, providing confidence in the routes derived. Additionally we were able to identify school entrances in an on-site audit improving the modelling of GIS routes.

However, limitations must also be acknowledged. Information on how each participant travelled to school on any given day were not generally available, so their self-reported usual mode of travel was used to determine GPS route mode and it is therefore likely that some routes were misclassified in terms of mode. Some data on actual route mode were available from the four-day food diary complete by SPEEDY participants, and which asked how the participant had travelled to and from school on two school days. In total 174 (99%) of the participants in these analyses completed the diary, and actual route mode was available for 464 of the 1191 GPS routes (39%). Of these 397 (86%) were made by the reported ‘usual’ mode of travel to school, as has been used in our analyses. This high agreement rate gives confidence to our findings, although the misclassification of route mode was not randomly distributed; of the 67 routes that were not made by the usual mode, 22 were journeys made on foot by children who reported usually travelling by car. This suggests that differences between car and walking routes may be underestimated in our models.

Only 8 of our participants reported usually travelling by bicycle. Although they provided 62 routes between them, numbers were still small, and so although differences between GIS and GPS routes for cyclists were detected, they were not statistically significant, possibly as a result of the small numbers.

To model routes in a GIS, defined start and end points are required, along with a network dataset. Home locations were derived from the address provided on the consent form (one address per participant), and we were therefore not able to account for instances where a child had more than one home. If a child had not travelled between school and the address on the consent form between the specified hours, the trip was not included in our analysis. This approach means that some legitimate routes to/from school may have been excluded.

The quality and completeness of the network used will impact the routes modelled. We were able to use a well-regarded, accurate road network for the modelling process, but this did not include footpaths or informal short-cuts. The overall median proportion of routes not on our road network was 0.3%, but was somewhat higher for pedestrians (4.8%). However, this may not give the complete picture of the impact the inclusion of footpaths may have on route modelling because the use of a small short-cut may only incur a small amount of travel ‘off-network’ but may enable a significantly different route to be taken, generating potentially large differences in environmental exposure.

The setting of the SPEEDY study within the county of Norfolk, UK may limit the transferability of our findings to other settings. Although we see no strong reason why the same factors would not impact GIS and GPS route differences in other similar settings (e.g. other rural counties in the UK or in other international settings), nor that some findings might have even wider transferability, care should be taken in assessing if and how the Norfolk situation may differ to other settings when attempting to apply these results in other contexts.

In conclusion, GIS modelled routes between home and school were not truly representative of accurate GPS measured exposure to obesogenic environments, particularly for pedestrians. While route length may be fairly well described, especially for urban populations, those living close to school, and those travelling by foot, the additional expense of acquiring GPS data, potentially coupled with behavioural surveys and interviews, seems important when assessing exposure to route environments.

## Competing interests

The authors have no competing interest to disclose.

## Authors’ contributions

FH developed the research question, processed and prepared GPS data, carried out the analyses, and drafted the manuscript. TB developed the research question, and processed and prepared GPS data. KC supervised and coordinated data collection. EvS and AJ were involved with the conceptualization and design of the SPEEDY study and supervised and coordinated data collection. All authors critically reviewed the manuscript, and approved the final manuscript as submitted.
